# MRT-Befundung hirneigener Tumoren: Was neuroonkologisch tätige Kliniker vom Radiologen erwarten

**DOI:** 10.1007/s00117-022-01014-6

**Published:** 2022-05-25

**Authors:** Torge Huckhagel, Christian Riedel

**Affiliations:** Institut für diagnostische und interventionelle Neuroradiologie, Universitätsmedizin Göttingen, Robert-Koch-Str. 40, 37075 Göttingen, Deutschland

**Keywords:** Hirntumoren, Neuroepitheliale Tumoren, Gliom, Magnetresonanztomographie, Strukturierte Befundung, Brain neoplasms, Neuroepithelial neoplasms, Glioma, Magnetic resonance imaging, Structured reporting

## Abstract

**Hintergrund und Ziel:**

Eine strukturierte MRT-Befundung unter Verwendung konsensbasierter inhaltlicher Kategorien hat das Potenzial, die interdisziplinäre Kommunikation in der Neuroonkologie zu verbessern. Ziel dieser Studie war es daher, mittels einer bundesweiten Befragung von Mitgliedern medizinischer Fachgesellschaften mit neuroonkologischem Bezug die wesentlichen Befundungskategorien der Bildgebung hirneigener Tumoren aus klinischer Perspektive zu ermitteln.

**Material und Methoden:**

Auf der Basis eines interdisziplinär entwickelten Katalogs von MRT-Befundungselementen wurde ein Online-Fragebogen erstellt. Im Anschluss wurden fachärztliche Mitglieder der Deutschen Gesellschaften für Neurochirurgie, Radioonkologie, Hämatologie und Medizinische Onkologie, Neurologie und Neuropathologie dazu eingeladen, die Items hinsichtlich ihrer klinischen Relevanz zu bewerten.

**Ergebnisse:**

An der Umfrage nahmen insgesamt 171 Fachärzte aus dem Bundesgebiet teil (81 Neurochirurgen, 66 Strahlentherapeuten und 24 andere neuroonkologische Experten). Anzahl und anatomische Ausdehnung der Tumoren in einer kontrastmittelverstärkten T1- und 2‑D-T2-Sequenz (98,8 % vs. 97,1 %) sowie neu diagnostizierte Läsionen bei Folgeuntersuchungen (T1 + Kontrast 98,2 %; T2 94,7 %) wurden am häufigsten als essenziell betrachtet. Darüber hinaus beurteilten die Experten insbesondere die Beschreibung einer ependymalen und/oder leptomeningealen Tumordissemination (93,6 %) sowie Zeichen der Raumforderung inklusive Verschlusshydrozephalus und parenchymale Massenverschiebungen (jeweils > 75,0 %) als wesentlich. Eine standardmäßige Erwähnung von intratumoralen Verkalkungen, Hämorrhagien, Tumorgefäßarchitektur oder erweiterter Bildgebungsmethoden wie MR-Perfusion, Diffusion, Traktographie und Protonenspektroskopie bewertete lediglich eine Minderheit der Umfrageteilnehmer als praxisrelevant.

**Schlussfolgerung:**

Ein zuweiserorientierter inhaltlicher Mindeststandard der magnetresonanztomographischen Hirntumordiagnostik sollte als klinisch relevante Kernelemente die exakte anatomische Ausbreitung der Raumforderung(en) inklusive ependymaler und meningealer Beteiligung sowie die einschlägigen Raumforderungszeichen enthalten.

## Abstract (en)

**Background and objective:**

Structured reporting of MRI examinations using consensus-based content categories has the potential to improve interdisciplinary communication in neuro-oncology. Therefore, the aim of this study was to determine the essential reporting categories in the imaging of gliomas from a clinical perspective within the setting of a nationwide survey of members of medical societies working in neuro-oncology.

**Materials and methods:**

An online questionnaire was created based on an interdisciplinary developed catalog of possible MRI reporting elements. Subsequently, specialist members of the German Societies of Neurosurgery, Radiation Oncology, Hematology and Medical Oncology, Neurology, and Neuropathology were invited to evaluate the items with regard to their clinical relevance.

**Results:**

A total of 171 specialists from Germany participated in the survey (81 neurosurgeons, 66 radiation therapists, and 24 other neuro-oncology experts). Number and anatomic extent of tumors in the contrast-enhanced T1 and 2D T2 sequences (98.8% vs. 97.1%) as well as newly diagnosed lesions at follow-up (T1 + contrast 98.2%; T2 94.7%) were overall most frequently considered crucial. In addition, the experts particularly rated the description of ependymal and/or leptomeningeal tumor dissemination (93.6%) and signs of mass effect including occlusive hydrocephalus and parenchymal mass shifts (> 75.0% each) as essential. Standard mention of intratumoral calcifications, hemorrhages, tumor vascular architecture, or advanced imaging modalities such as MR perfusion, diffusion, tractography, and proton spectroscopy were considered fundamental to their everyday practice by only a minority of neuro-oncology colleagues.

**Conclusion:**

A referring physician-oriented minimum content standard for MRI examinations in primary brain tumor patients should include as clinically relevant core elements the exact anatomic spread of the lesion(s), including ependymal and meningeal involvement, and the pertinent signs of mass effect.

Gliome repräsentieren den größten Anteil der primären intrakraniellen Tumoren. Während diese Neoplasien einerseits relativ selten auftreten, sind sie andererseits mit einer signifikanten Morbidität und Mortalität vergesellschaftet. Im Kontext der interdisziplinären medizinischen Versorgung von Hirntumorpatienten stellt der radiologische Befundbericht ein zentrales Kommunikationselement dar. Um dieser Aufgabe optimal gerecht zu werden, sollte er sich inhaltlich am spezifischen Informationsbedarf der zuweisenden ärztlichen Kollegen orientieren. In diesem Beitrag werden die Ergebnisse einer bundesweiten Expertenumfrage vorgestellt, im Rahmen derer der Informationsbedarf für MRT-Befunde von Patienten mit Gliomen ermittelt wurde.

Das Zentrale Hirntumorregister der Vereinigten Staaten von Amerika beziffert die durchschnittliche jährliche altersadjustierte Gesamtinzidenz benigner und maligner primärer Tumoren des zentralen Nervensystems (ZNS) auf 28,57 Fälle pro 100.000 Einwohner [10]. Den größten Anteil an den primären Hirntumoren machen hierbei die Gliome mit einer altersadjustierten jährlichen Gesamtinzidenz zwischen 4,67 und 5,73 Fällen pro 100.000 Einwohner aus. Das Glioblastom, dem nach der aktuellen 5. Edition der WHO-Klassifikation der Tumoren des ZNS in Kontinuität zu den vorherigen Ausgaben der höchste Malignitätsgrad (WHO Grad 4) zugeordnet wird, bildet unter den Gliomen wiederum im Erwachsenenalter die häufigste Entität mit der schlechtesten Prognose [8, 9]. Patienten, die an einem Glioblastom erkranken, weisen unter der etablierten Standardbehandlung mit konkomitanter Radiochemotherapie (Gesamtdosis 60 Gy; 30 Fraktionen) und nachfolgender adjuvanter Fortführung der temozolomidbasierten Chemotherapie über 6 Zyklen (sog. Stupp-Protokoll) ein medianes Gesamtüberleben von 14,6 Monaten auf [11]. Betrachtet man hingegen pädiatrische und adoleszente Patienten (< 20 Jahre), so sind ZNS-Tumoren in dieser Altersgruppe die häufigsten Malignome, gefolgt von hämatologischen Neoplasien [10]. Bei der neuroradiologischen Beschreibung hirneigener Tumoren wurden in jüngerer Vergangenheit Versuche einer strukturierten Befunderhebung unternommen, wie sie bereits seit längerer Zeit in anderen radiologischen Subdisziplinen erfolgt sind. Wesentliche Limitation der bisherigen Studien ist dabei allerdings, dass die jeweiligen vorgestellten Befundungsmuster entweder lediglich monodisziplinär von Neuroradiologen oder monozentrisch entwickelt bzw. implementiert wurden und somit nicht ohne Weiteres als repräsentativ und dem Bedarf der klinischen zuweisenden Fächer angemessen angesehen werden können [1, 5]. Wir haben uns daher zum Ziel gesetzt, im Rahmen einer bundesweiten Online-Befragung unter neuroonkologisch tätigen Fachärzten verschiedener medizinischer Disziplinen zu erheben, welche Befundungselemente bei der Evaluation von MRT-Untersuchungen von Patienten mit hirneigenen Tumoren aus ihrer spezifischen klinischen Perspektive inhaltlich relevant sind und daher in einem radiologischen Befundbericht grundsätzlich repräsentiert sein sollten.

## Material und Methoden

### Ethik und Studiendesign

Das Studienprotokoll wurde vorab von der zuständigen institutionellen Ethikkommission der Universitätsmedizin Göttingen beraten und genehmigt (Antragsnummer 9/2/21), wobei es sich bei dem Vorhaben formal um kein medizinisches Forschungsprojekt am Menschen handelt und somit keine grundsätzliche Beratungspflicht durch eine medizinische Ethikkommission besteht. Das Studiendesign umfasst eine bundesweite strukturierte Online-Umfrage unter medizinischen Spezialisten der neuroonkologischen Patientenversorgung mit Facharztstatus im Sinne einer prospektiven Querschnittserhebung. Die Präsentation der Daten folgt, wo immer dies möglich ist, den Empfehlungen von Kelley und Kollegen zur guten Praxis in der Durchführung und Berichterstattung von Umfrageforschung [6].

### Studienablauf

**Figure 1:**
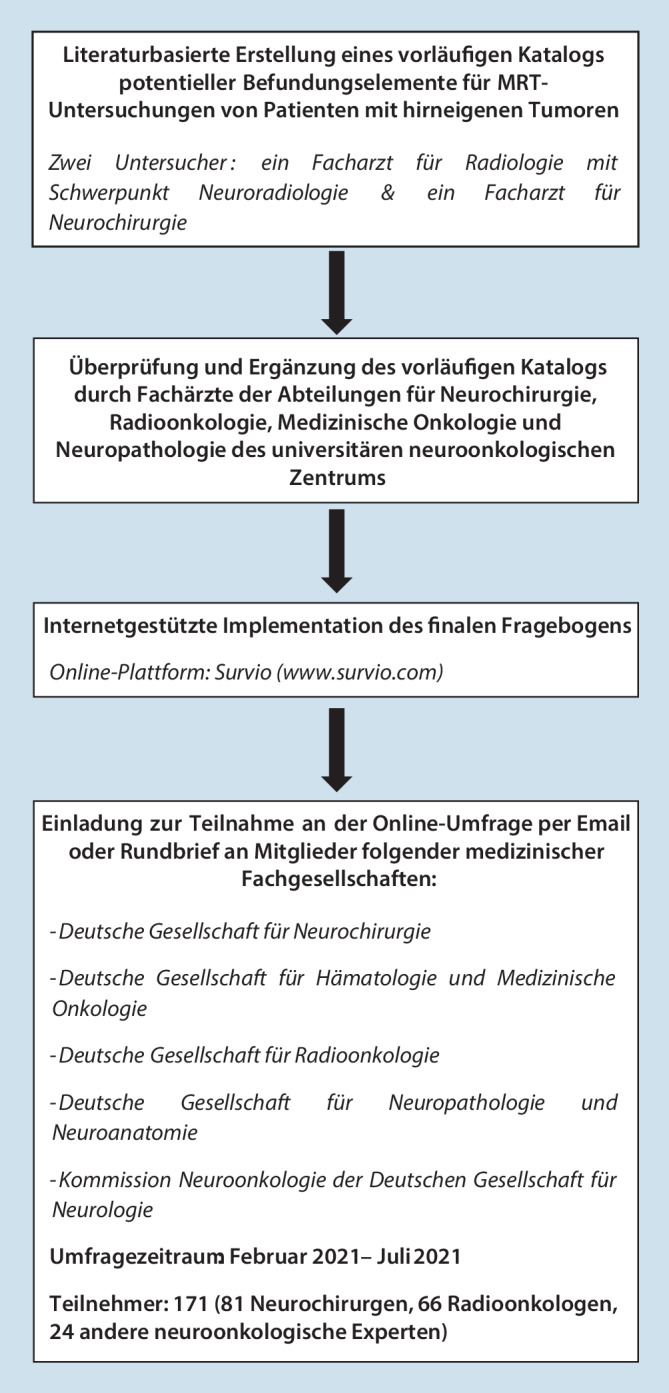

Anfänglich erfolgte eine umfassende Zusammenstellung potenzieller Befundungselemente für MRT-Untersuchungen an Patienten mit hirneigenen Tumoren. Hierbei wurden Informationen aus der einschlägigen internationalen englisch- und deutschsprachigen neuroradiologischen und neurochirurgischen Fachliteratur integriert und um Erfahrungen der Autoren, eines Facharztes für Radiologie mit Schwerpunkt Neuroradiologie und eines Facharztes für Neurochirurgie, ergänzt. Im Anschluss daran befragten wir systematisch fachärztliche Kollegen aus den Disziplinen Neuroradiologie, Neurochirurgie, Strahlentherapie, Medizinische Onkologie, Neurologie und Neuropathologie an unserem Universitäts-Krebszentrum, um zum einen die Verständlichkeit bzw. Eindeutigkeit der gelisteten Elemente zu überprüfen und zum anderen weitere Ergänzungsvorschläge einzuholen. Der erweiterte Katalog umfasste 28 Einträge und wurde als finaler Online-Fragebogen auf der Plattform Survio (www.survio.com) implementiert. Für jedes einzelne Element wurden die Befragten im Rahmen der Studie darum gebeten, aus ihrer persönlichen klinischen Perspektive heraus zwischen den beiden Optionen „wesentlich“ und „unwesentlich“ für den MRT-Befund zu wählen. Durch die Unterstützung der Deutschen Gesellschaft für Neurochirurgie (www.dgnc.de), der Deutschen Gesellschaft für Radioonkologie (www.degro.org) und der Klinischen Kommission Neuroonkologie der Deutschen Gesellschaft für Neurologie (www.dgn.org) konnten alle registrierten fachärztlichen Mitglieder dieser Institutionen über die entsprechenden E‑Mail-Verteiler persönlich zur Teilnahme an dem Projekt eingeladen werden. Mitglieder der Deutschen Gesellschaft für Hämatologie und Medizinische Onkologie (www.dgho.de) wurden in Form eines Newsletters der Fachgesellschaft zur Abstimmung aufgerufen. Zusätzlich nahmen wir zu sämtlichen auf der Webseite der Deutschen Gesellschaft für Neuropathologie und Neuroanatomie (www.dgnn.de) vertretenen Einrichtungen und Instituten in der Bundesrepublik Deutschland per E‑Mail Kontakt auf und luden zur Teilnahme an dem Projekt ein. Die Mitglieder der genannten Fachgesellschaften wurden darum gebeten, nur im Falle vorliegender eigener praktischer Erfahrungen in der Behandlung von Hirntumorpatienten an der Umfrage teilzunehmen. Eine Übersicht zum Studienablauf ist Abb. 1 (Studiendesign) zu entnehmen.

### Studienteilnehmer

Insgesamt wurde der Fragebogen von 171 Fachärzten vollständig ausgefüllt eingereicht. Die Möglichkeit zur Teilnahme an der Erhebung umfasste den Zeitraum vom 19.02.2021 bis einschließlich zum 01.07.2021 (132 Tage). Die zur Bearbeitung der Fragen benötigte Zeit betrug in der Mehrheit der Fälle weniger als 10 min (82,4 %). Unter den 171 Teilnehmern befanden sich 81 Neurochirurgen, 66 Strahlentherapeuten/Radioonkologen und 24 andere Experten aus den Gebieten der medizinischen Onkologie und Neuropathologie.

### Statistik

Die kumulativen Umfrageergebnisse werden als numerische Verhältnisse (Anzahl der Positivbewertungen/Anzahl der Studienteilnehmer) bzw. prozentuale Zustimmungswerte präsentiert. Für klinisch bedeutsame Ergebnisse werden ergänzend 95 %-Konfidenzintervalle (KI) als Streuungsmaß angegeben. Zusätzlich haben wir für die einzelnen MRT-Befundungselemente einen Gruppenvergleich zwischen Neurochirurgen, Strahlentherapeuten/Radioonkologen und anderen Experten (medizinische Onkologen, Neuropathologen, Neurologen mit neuroonkologischem Schwerpunkt) vorgenommen. Zur Bestimmung signifikanter Gruppenunterschiede hinsichtlich der Präferenz einzelner potenzieller Items wurde der Chi-Quadrat-Test eingesetzt (Signifikanzniveau 0,05).

## Ergebnisse

### Kumulative Bewertung der MRT-Befundungselemente

**Figure 2:**
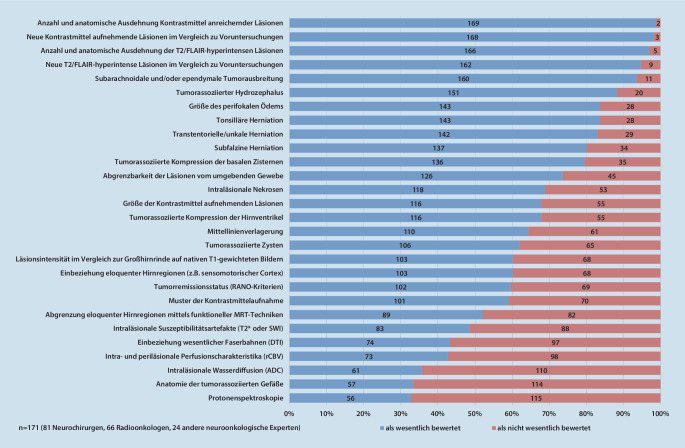

Als wichtigste Inhaltspunkte für die MRT-Befundung hirneigener Tumoren nannten die Studienteilnehmer einerseits die Anzahl und detaillierte neuroanatomische Ausdehnung der zerebralen Raumforderung(en) in der kontrastmittelgestützten T1-Sequenz (169/171; 98,8 %; KI 84,5–100,0 %) und in der T2- bzw. FLAIR-Sequenz (166/171; 97,1 %; KI 82,9–100 %) sowie andererseits im Verlauf neu aufgetretene Kontrastmittel aufnehmende (168/171; 98,2 %; KI 83,9–100,0 %) und T2/FLAIR-hyperintense Läsionen (162/171; 94,7 %; KI 80,7–100,0 %) im Vergleich zu MRT-Voruntersuchungen des betreffenden Patienten. Weiterhin wurde die explizite Erwähnung einer ependymalen und/oder leptomeningealen Tumordissemination als besonders wichtig eingestuft (160/171; 93,6 %; KI 79,6–100,0 %). Darüber hinaus erachteten über 75 % der Studienteilnehmer solche Parameter als essenzielle inhaltliche Bestandteile eines MRT-Befunds, die unmittelbare Folge der Raumforderung des Tumors selbst und/oder seines perifokalen Ödems sind. Hierzu gehören insbesondere der Hydrocephalus occlusus und die parenchymalen Massenverschiebungen: subfalzine Herniation, transtentorielle Herniation (sog. *obere Einklemmung*) und Kleinhirntonsillenherniation im Foramen magnum (sog. *untere Einklemmung*). Zwischen 50 und 75 % der Befragten befanden spezifische MRT-Charakteristika der Raumforderung wie die Abgrenzbarkeit gegenüber dem umliegenden Hirngewebe, intraläsionale Nekrosen und Zysten, das Kontrastmittelanreicherungsmuster und die exakte Größe des schrankengestörten Tumoranteils sowie die Deskription des Therapieansprechens mittels der für klinische Studien etablierten RANO-Kriterien (Response Assessment in Neuro-Oncology) als essenziell. Außerdem würde die Mehrheit der Experten die explizite Beschreibung einer tumorbedingten Pelottierung der inneren Liquorräume, einer Verschiebung der Mittellinie sowie einer Einbeziehung eloquenter Hirnregionen (z. B. sensomotorischer Kortex, primäre Sehrinde, Sprachareale) in das Tumorwachstum begrüßen. Zur Beurteilung der letztgenannten Kategorie wünschten sich die Befragten mehrheitlich auch eine ergänzende funktionelle MRT-Diagnostik, sofern in Zusammenschau des klinischen Befunds und der konventionellen Bildgebung diese Frage nicht adäquat zu beantworten ist. Die explizite Nennung von Tumorverkalkungen, Einblutungen, der makroskopisch sichtbaren Tumorgefäßarchitektur und erweiterter Bildgebungsmethoden inklusive Traktographie, MR-Spektroskopie sowie Perfusions- und Diffusionscharakteristika wurde lediglich von einer Minderheit (< 50 %) der neuroonkologisch tätigen befragten Kollegen als relevant für ihre jeweilige tägliche Praxis beschrieben. Eine detaillierte Aufstellung der kumulativen Bewertung sämtlicher untersuchter Befundungsitems ist Abb. 2 (Gesamtbewertung) zu entnehmen.

### Interdisziplinäre Unterschiede in der Bewertung der MRT-Befundungselemente

Bei der Hälfte (14/28) der untersuchten potenziellen Befundungskategorien differierten die Einschätzungen zwischen Neurochirurgen, Strahlentherapeuten/Radioonkologen und anderen neuroonkologisch tätigen Experten signifikant (*p* < 0,05). Eine Übersicht hinsichtlich der unterschiedlichen Präferenzen der einzelnen Disziplinen bietet Tab. 1 (Gruppenspezifische Präferenzen). Die detaillierten Einzelbewertungen aller Befundungselemente für jede Expertengruppe finden sich im Tab. 2 (Gruppenspezifische Bewertung). Während für Neurochirurgen und Strahlentherapeuten/Radioonkologen vor allem die Beschreibung einer subarachnoidalen und/oder ependymalen Tumorausbreitung sowie im Verlauf neu auftretende bzw. sich vergrößernde T2-/FLAIR-hyperintense Läsionen von herausragender Bedeutsamkeit waren (für beide Gruppen jeweils Zustimmungsraten > 95,0 %), maßen die Experten der medizinischen Onkologie und Neuropathologie diesen Faktoren eine vergleichsweise geringere Wertigkeit zu (subarachnoidales/ependymales Wachstum 75,0 %; neue T2-/FLAIR-hyperintense Läsionen im Verlauf 83,3 %). Der Nennung von Raumforderungszeichen inklusive einer Mittellinienverlagerung und Kompression der inneren bzw. äußeren Liquorräume sowie parenchymaler Massenverschiebungen (subfalzine und transtentorielle Herniation) hingegen wurde tendenziell von Fachärzten der nichtchirurgischen Disziplinen im Vergleich zu den befragten Neurochirurgen eine höhere Priorität zugeordnet. So wurde exemplarisch die Mittellinienverlagerung als etabliertes Kriterium einer intrakraniellen Massenverschiebung von 80,3 % der Strahlentherapeuten/Radioonkologen und 66,7 % der anderen nichtchirurgisch tätigen neuroonkologischen Experten als wesentliches Befundelement angeführt, während die Zustimmung von neurochirurgischer Seite mit 50,6 % deutlich geringer ausfiel. Die Erwähnung intraläsionaler Nekrosen wurde insbesondere von den Kollegen der Neuropathologie und medizinischen Onkologie als essenziell eingestuft (91,7 %).

**Table 1:** 

Befundungselement	Neurochirurgen (*n* = 81)	Radioonkologen (*n* = 66)	Andere Experten (*n* = 24)	*p*-Wert*
Subarachnoidale und/oder ependymale Tumorausbreitung	++++	++++	++	< 0,01
Größe der Kontrastmittel anreichernden Läsionen	++	+++	+++	0,01
Muster der Kontrastmittelaufnahme	++	+	++	< 0,01
Neue T2/FLAIR-hyperintense Läsionen im Vergleich zu Voruntersuchungen	++++	++++	+++	0,03
Intraläsionale Suszeptibilitätsartefakte (T2* oder SWI)	++	+	++	< 0,01
Einbeziehung wichtiger Faserbahnen (DTI)	++	+	−	< 0,01
Intraläsionale Nekrosen	++	++	+++	0,03
Anatomie der tumorassoziierten großen Gefäße	+	−	−	< 0,01
Mittellinienverlagerung	++	+++	++	< 0,01
Tumorassoziierte Kompression der Hirnventrikel	+	+++	++	< 0,01
Tumorassoziierte Kompression der basalen Zisternen	++	++++	++	< 0,01
Subfalzine Herniation	++	+++	+++	< 0,01
Transtentorielle/unkale Herniation	++	+++	+++	0,01
Tonsilläre Herniation	+++	+++	+++	0,01

Zustimmungsraten von x ≤ 25 % sind als (−) dargestellt, 25 % < x ≤ 50 % entsprechen (+), 50 % < x ≤ 75 % entsprechen (++), 75 % < x ≤ 95 % entsprechen (+++), and x > 95 % entsprechen (++++)

*DTI* „diffusion tensor imaging“, *FLAIR* „fluid-attenuated inversion recovery“, *SWI* „susceptibility weighted imaging“

*Chi-Quadrat-Test (Signifikanzniveau 0,05)

**Table 2:** 

Befundungselement	Neurochirurgen	Radioonkologen	Andere Experten	Gesamt	*p*-Wert*
Anzahl und anatomische Ausdehnung der T2/FLAIR-hyperintensen Läsionen (*n*/*N*; %)	79/81 (97,5)	64/66 (97,0)	23/24 (95,8)	166/171 (97,1)	0,91
Läsionsintensität im Vergleich zur Großhirnrinde auf nativen T1-gewichteten Bildern (*n*/*N*; %)	49/81 (60,5)	37/66 (56,1)	17/24 (70,8)	103/171 (60,2)	0,45
Anzahl und anatomische Ausdehnung kontrastmittelanreichernder Läsionen (*n*/*N*; %)	80/81 (98,8)	65/66 (98,5)	24/24 (100,0)	169/171 (98,8)	0,84
Subarachnoidale und/oder ependymale Tumorausbreitung (*n*/*N*; %)	77/81 (95,1)	65/66 (98,5)	18/24 (75,0)	160/171 (93,6)	**<** **0,01**
Größe der kontrastmittelanreichernden Läsionen (*n*/*N*; %)	46/81 (56,8)	51/66 (77,3)	19/24 (79,2)	116/171 (67,8)	**0,01**
Muster der Kontrastmittelaufnahme (*n*/*N*; %)	56/81 (69,1)	28/66 (42,4)	17/24 (70,8)	101/171 (59,1)	**<** **0,01**
Abgrenzbarkeit der Läsionen vom umgebenden Gewebe (*n*/*N*; %)	57/81 (70,4)	50/66 (75,8)	19/24 (79,2)	126/171 (73,7)	0,61
Größe des perifokalen Ödems (*n*/*N*; %)	68/81 (84,0)	57/66 (86,4)	18/24 (75,0)	143/171 (83,6)	0,43
Neue kontrastmittelaufnehmende Läsionen im Vergleich zu Voruntersuchungen (*n*/*N*; %)	78/81 (96,3)	66/66 (100,0)	24/24 (100,0)	168/171 (98,2)	0,18
Neue T2/FLAIR*-hyperintense Läsionen im Vergleich zu Voruntersuchungen (*n*/*N*; %)	78/81 (96,3)	64/66 (97,0)	20/24 (83,3)	162/171 (94,7)	**0,03**
Einbeziehung eloquenter Hirnregionen (z. B. sensomotorischer Kortex) (*n*/*N*; %)	51/81 (63,0)	37/66 (56,1)	15/24 (62,5)	103/171 (60,2)	0,68
Intraläsionale Suszeptibilitätsartefakte (T2* oder SWI) (*n*/*N*; %)	48/81 (59,3)	19/66 (28,8)	16/24 (66,7)	83/171 (48,5)	**<** **0,01**
Intraläsionale Wasserdiffusion (ADC) (*n*/*N*; %)	30/81 (37,0)	18/66 (27,3)	13/24 (54,2)	61/171 (35,7)	0,06
Einbeziehung wichtiger Faserbahnen (DTI) (*n*/*N*; %)	51/81 (63,0)	18/66 (27,3)	5/24 (20,8)	74/171 (43,3)	**<** **0,01**
Intraläsionale Nekrosen (*n*/*N*; %)	53/81 (65,4)	43/66 (65,2)	22/24 (91,7)	118/171 (69,0)	**0,03**
Tumorassoziierte Zysten (*n*/*N*; %)	49/81 (60,5)	38/66 (57,6)	19/24 (79,2)	106/171 (62,0)	0,16
Intra- und periläsionale Perfusionscharakteristika (RCBV) (*n*/*N*; %)	41/81 (50,6)	21/66 (31,8)	11/24 (45,8)	73/171 (42,7)	0,07
Anatomie der tumorassoziierten großen Gefäße (*n*/*N*; %)	39/81 (48,1)	12/66 (18,2)	6/24 (25,0)	57/171 (33,3)	**<** **0,01**
Tumorremissionsstatus (RANO-Kriterien) (*n*/*N*; %)	43/81 (53,1)	41/66 (62,1)	18/24 (75,0)	102/171 (59,6)	0,14
Mittellinienverlagerung (*n*/*N*; %)	41/81 (50,6)	53/66 (80,3)	16/24 (66,7)	110/171 (64,3)	**<** **0,01**
Tumorassoziierte Kompression der Hirnventrikel (*n*/*N*; %)	39/81 (48,1)	60/66 (90,9)	17/24 (70,8)	116/171 (67,8)	**<** **0,01**
Tumorassoziierte Kompression der basalen Zisternen (*n*/*N*; %)	55/81 (67,9)	63/66 (95,5)	18/24 (75,0)	136/171 (79,5)	**<** **0,01**
Tumorassoziierter Hydrozephalus (*n*/*N*; %)	69/81 (85,2)	62/66 (93,9)	20/24 (83,3)	151/171 (88,3)	0,19
Subfalzine Herniation (*n*/*N*; %)	56/81 (69,1)	61/66 (92,4)	20/24 (83,3)	137/171 (80,1)	**<** **0,01**
Transtentorielle/unkale Herniation (*n*/*N*; %)	60/81 (74,1)	62/66 (93,9)	20/24 (83,3)	142/171 (83,0)	**0,01**
Tonsilläre Herniation (*n*/*N*; %)	61/81 (75,3)	62/66 (93,9)	20/24 (83,3)	143/171 (83,6)	**0,01**
Protonenspektroskopische Tumorcharakteristika (*n*/*N*; %)	30/81 (37,0)	19/66 (28,8)	7/24 (29,2)	56/171 (32,7)	0,53
Abgrenzbarkeit eloquenter Hirnregionen mittels funktioneller MRT-Techniken (*n*/*N*; %)	48/81 (59,3)	31/66 (47,0)	10/24 (41,7)	89/171 (52,0)	0,18

Diese Tabelle zeigt die anteilige Befürwortung der Integration spezifischer MRT-Bildbefunde in den radiologischen Bericht bei Gliompatienten seitens verschiedener medizinischer Disziplinen (81 Neurochirurgen, 66 Radioonkologen sowie 24 andere Experten aus den Bereichen medizinische Onkologie/Neuroonkologie und Neuropathologie). Die Umfrageergebnisse sind als Fraktion (*n/N*;* n* = Anzahl der Befürworter aus der jeweiligen Disziplin; *N* = Gesamtzahl der Befragten der jeweiligen Disziplin) und prozentual angegeben.

*ADC* „apparent diffusion coefficient“, *DTI* „diffusion tensor imaging“, *FLAIR* „fluid-attenuated inversion recovery“, *RANO* Response Assessment in Neuro-Oncology, *RCBV* „relative cerebral blood volume“, *SWI* „susceptibility weighted imaging“

*Chi-Quadrat-Test (Signifikanzniveau 0,05)

## Diskussion

Im Rahmen der hier vorliegenden Studie wurde unserer Kenntnis nach erstmalig auf Grundlage einer interdisziplinären Befragung von Neurochirurgen, Strahlentherapeuten/Radioonkologen, medizinischen Onkologen und Neuropathologen auf nationaler Ebene ein inhaltlicher bedarfsorientierter Mindeststandard für die MRT-Befundung hirneigener Tumoren erarbeitet. Es zeigte sich hierbei, dass für neuroonkologisch tätige Kliniker insbesondere die Anzahl und anatomische Ausdehnung der Tumoren unter besonderer Berücksichtigung einer möglichen ependymalen und/oder leptomeningealen Tumoraussaat, das Auftreten neuerlicher Läsionen bei Verlaufsuntersuchungen sowie auch Folgen der Raumforderung wie parenchymale Massenverschiebungen oder ein Verschlusshydrozephalus von herausragender Bedeutung sind. Über diesen Mindeststandard hinaus möchten wir dem Radiologen zudem eine praktikable Handreichung dafür geben, wie er seine Befundberichte zielgerichtet dem Informationsbedarf seiner Zuweiser entsprechend erweitern kann. Hierfür legen wir eine nach den Präferenzen der neuroonkologischen Disziplinen geordnete Rangfolge der prinzipiell möglichen Befundungskategorien als Orientierungshilfe vor. Für die Beschreibung der anatomischen Ausdehnung der Tumoren wie auch die spezifischen strukturellen Charakteristika der Raumforderungen (u. a. Nekrosen, Zysten, Kontrastmittelanreicherungsmuster, Abgrenzbarkeit gegenüber dem Hirnparenchym) bietet sich in erster Linie das VASARI-Lexikon an (Visually Accessible Rembrandt Images Research Project; [12]). Diese standardisierte Terminologie repräsentiert derzeit das am weitesten entwickelte System zur qualitativen Deskription von MRT-Merkmalen bei Gliomen, wobei für bestimmte VASARI-Parameter (insbesondere Multifokalität bzw. Multizentrizität) auch ein prognostischer Wert zusätzlich zum molekularen Tumorprofil und klinischen Risikofaktoren der Patienten nachgewiesen werden konnte [7]. Die grundsätzliche Vorgehensweise, ein Produkt oder eine Dienstleistung an den Bedürfnissen und Wünschen der Verbraucher bzw. Kunden auszurichten, ist in den meisten marktwirtschaftlich orientierten Industrien und Gewerben eine bewährte Praxis. In der Radiologie wurden bislang jedoch nur selten Anstrengungen unternommen, die erbrachte *Dienstleistung* des radiologischen Befundberichts gemäß den Präferenzen der zuweisenden klinischen Fächer zu optimieren, sodass dieser seinen größtmöglichen Nutzen im Sinne der Patientenversorgung entfalten kann. Eine der wenigen Studien auf diesem Gebiet evaluierte monozentrisch die Haltung und Einstellung der zuweisenden medizinischen Disziplinen gegenüber der radiologischen Befunderstellung und Nachverarbeitung von bildgebenden Untersuchungen anhand der sog. Kundenbedürfnismethode, welche im angelsächsischen Sprachraum als „voice-of-the-customer method“ bekannt ist [2]. Die Analyse der gewonnenen Daten zeigte, dass die größten von den klinisch tätigen Kollegen wahrgenommenen Defizite darin bestehen, dass ihr spezifischer Informationsbedarf nicht genügend berücksichtigt und praxisrelevante Schlüsselinformationen im Befundbericht nicht hinreichend kommuniziert werden. In der jüngeren Vergangenheit gab es allerdings bereits initiale Unternehmungen an der Emory Universitätsklinik in Atlanta (USA), eine zuweiserorientierte MRT-Befundung von Hirntumoren zu etablieren [15]. Das Befundungsmuster des Brain Tumor Reporting and Data System (BT-RADS) umfasst hierbei in Übereinstimmung mit unseren Ergebnissen die exakte Lokalisation des Tumors, die Ausdehnung der FLAIR-hyperintensen Alterationen des Hirnparenchyms, neue FLAIR-Läsionen bei Verlaufsuntersuchungen, die Ausdehnung des KM-aufnehmenden Tumoranteils sowie neuerliche Schrankenstörungen bei Verlaufsuntersuchungen [3]. Eine Diskrepanz besteht hingegen im Hinblick auf andere BT-RADS-Elemente, wie tumorassoziierte Diffusionseinschränkungen und – allerdings auch hier nur optional zu integrierende – Perfusions- und Protonenspektroskopie-Befunde. Die Mehrheit der im Rahmen unserer bundesweiten Studie befragten Experten bewertete diese Items als nicht wesentlich für ihre tägliche neuroonkologische Praxis. In einer klinikinternen Folgebefragung wurde den amerikanischen Kollegen aus Atlanta nach weitgehender Implementation des BT-RADS-Befundungsstandards eine deutliche Qualitätsverbesserung der radiologischen Berichte bezüglich Kohärenz, Eindeutigkeit und interdisziplinärer Kommunikation und damit einhergehend eine höhere Gesamtzufriedenheit seitens der überweisenden ärztlichen Kollegen bestätigt [5]. Zudem konnte eine signifikante Verkürzung der Befundtexte ohne Verlust relevanter Informationen erreicht werden [17]. Diese Ergebnisse werden durch eine weitere Studie aus der Schweiz untermauert, in der die Kollegen zeigen konnten, dass eine strukturierte MRT-Befundung intrakranieller Tumoren im Vergleich zur konventionellen Freitextbefundung bei ähnlichem Zeitaufwand einen höheren Informationsgehalt bietet [1]. Das hierbei zugrundegelegte, von Neuroradiologen entwickelte strukturierte Befundungsmuster weist deutliche Parallelen bezüglich der tumorassoziierten Befundungselemente zu unseren Ergebnissen auf und integriert im Vergleich zum BT-RADS deutlich mehr Details. So werden hier neben der Anzahl, anatomischen Lokalisation und Ausdehnung (T2/FLAIR/T1 nach Kontrastmittelapplikation) der Tumoren auch u. a. die Einbeziehung eloquenter Hirnregionen, raumfordernde Effekte (z. B. Mittellinienverlagerung, Ventrikelkompression, Hydrocephalus occlusus) und Einblutungen gesondert beschrieben. In der Zusammenschau erlauben die zitierten Arbeiten die Schlussfolgerung, dass durch die Anwendung einer strukturierten Befundungsmethodik einerseits und die inhaltliche Ausrichtung an den Informationsbedürfnissen der überweisenden Fachdisziplinen andererseits ein positiver Synergismus im Hinblick auf die interdisziplinäre Kommunikation und damit einhergehend auch auf die neuroonkologische Patientenversorgung erreicht werden kann. Beide vorgestellten Befundungsmuster und auch die Ergebnisse aus unserer Befragung gehen mit den MRT-Kriterien der RANO-Arbeitsgruppe konform, welche einen Standard für die Evaluation des Therapieansprechens hirneigener Tumoren in klinischen Studien etablieren und darüber hinaus auch für die klinische Praxis empfohlen werden [4, 16]. Die bildgebenden RANO-Kriterien für maligne Gliome beinhalten, unter Berücksichtigung des klinischen Patientenzustands und der Kortikosteroidmedikation, die prozentuale Zu- oder Abnahme des kontrastmittelaufnehmenden Tumors (zweidimensionale Messung), die Entwicklung der T2-/FLAIR-hyperintensen Parenchymalterationen und das Auftreten neuer Läsionen bei MRT-Verlaufsuntersuchungen. Für niedriggradige hirneigene Tumoren existieren modifizierte Kriterien, bei denen die prozentuale Veränderung der T2-/FLAIR-Signalalterationen des Hirngewebes im zeitlichen Verlauf im Vordergrund steht. Die Studienteilnehmer äußerten ein vergleichsweise geringes Bedürfnis nach expliziter Anwendung der RANO-Kriterien im Befundbericht (59,6 % Gesamtzustimmungsrate). Dies ist dadurch erklärlich, dass anhand der mit einer deutlichen Mehrheit gewünschten Beschreibung der Anzahl und anatomischen Ausdehnung der Tumoren in der kontrastmittelgestützten T1- und der T2/FLAIR-Sequenz sowie der Charakterisierung von im Verlauf neu auftretenden Läsionen unter Hinzuziehung des klinischen Zustands und der aktuellen Steroidmedikation des jeweiligen Patienten der RANO-Status problemlos bestimmt werden kann. Eine zusätzliche Erwähnung im radiologischen Befundbericht erscheint somit aus Gründen der Redundanz nicht zwingend notwendig und im Falle unvollständiger anamnestischer Angaben zu den genannten klinischen Parametern auch nicht sicher möglich. Interessanterweise wurden erweiterte Methoden der Bildgebung wie Diffusion, Protonenspektroskopie und MR-Perfusion von Neurochirurgen, Strahlentherapeuten/Radioonkologen und anderen neuroonkologischen Experten als nachrangig für ihre jeweilige klinische Praxis eingestuft. Während unter dem Gesichtspunkt des Informationsbedürfnisses der ärztlichen Zuweiser eine Integration dieser Informationen in den radiologischen Befund also nicht zwingend notwendig wäre, so haben doch zahlreiche Studien in der Vergangenheit einen Zusatznutzen dieser weiterführenden Verfahren in der Hirntumordiagnostik belegen können, sodass der Radiologe bei der Bildinterpretation hierauf nicht verzichten sollte. Insbesondere die Protonenspektroskopie und die MR-Perfusion sind hierbei wertvolle Instrumente für die Abgrenzung von niedriggradigen gegenüber malignen Gliomen und die Differenzierung zwischen vitalem Tumorgewebe und einer reaktiven Schrankenstörung im Sinne einer Pseudoprogression nach Radiochemotherapie [13, 14]. Aufgrund der differenzialdiagnostischen Evidenz und der damit verbundenen therapeutischen Konsequenz der genannten neueren MRT-Techniken sollten diese bei entsprechender Fragestellung als wertvolle Befundinterpretationshilfen einbezogen werden und Eingang in die abschließende radiologische Beurteilung finden, auch wenn diesbezügliche Ausführungen im beschreibenden Teil des Befundberichts seitens der Zuweiser nicht mehrheitlich gefordert werden.

### Stärken und Schwächen

Eine wesentliche Stärke dieser Studie liegt darin begründet, dass durch die Unterstützung der entsprechenden medizinischen Fachgesellschaften erstmalig eine bundesweit angelegte interdisziplinäre Umfrage zur Ermittlung eines an den Zuweisern orientierten inhaltlichen Mindeststandards in der MRT-Befundung hirneigener Tumoren durchgeführt werden konnte. Während hierdurch die Einschränkungen bisheriger Studien hinsichtlich ihres monozentrischen und/oder monodisziplinären Ansatzes überwunden wurden, besteht bei einer a priori generierten Liste potenzieller Befundungselemente noch das grundsätzliche Risiko einer möglichen Unvollständigkeit. Um diesem Problem zu begegnen, war es den befragten Experten möglich, Ergänzungen im Freitextformat einzubringen. Die hier unterbreiteten Vorschläge waren jedoch sämtlich Einzelmeinungen und damit nicht repräsentativ, sodass eine Integration dieser Zusätze in einen MRT-Befundungsstandard nicht gerechtfertigt erscheint. Ein Vergleich der Teilnehmerzahl mit den Mitgliedern der involvierten Fachgesellschaften offenbart eine relative Unterrepräsentation der medizinischen Neuroonkologen. Während ungefähr 5 % der registrierten Mitglieder der Deutschen Gesellschaft für Neurochirurgie (81/1550 Personen; Stand August 2021) und der Deutschen Gesellschaft für Radioonkologie (66/1326 Personen; Stand August 2021) an der Befragung teilgenommen haben, lag die Beteiligungsrate der Mitglieder der Deutschen Gesellschaft für Hämatologie und Medizinische Onkologie mit ca. 0,5 % deutlich niedriger (16/~3500 Personen). Dieses Ungleichgewicht zugunsten der Vertreter anatomisch zielgerichteter Therapien birgt grundsätzlich das Risiko eines Bias in sich, welches bei der Interpretation der kumulativen Ergebnisse berücksichtigt werden sollte. Um dem potenziellen Problem der Stichprobenverzerrung zu begegnen, wurden die Präferenzen der verschiedenen Fachdisziplinen einander gegenübergestellt und systematisch verglichen (Tab. 1 und 2).

## Ausblick

Die Implementation einer strukturierten MRT-Befundung anhand eines an den Bedürfnissen der klinischen Zuweiser orientierten inhaltlichen Standards bietet für die radiologische Hirntumordiagnostik das Potenzial, die interdisziplinäre Kommunikation und im Zusammenhang damit auch die neuroonkologische Patientenversorgung positiv zu beeinflussen. Ein Mindeststandard sollte hierbei als Kernelemente insbesondere die exakte anatomische Ausbreitung der Tumoren inklusive ependymaler und meningealer Beteiligung sowie die einschlägigen Zeichen der Raumforderung enthalten. Die Ergebnisse der vorliegenden Expertenbefragung bieten darüber hinaus eine bedarfsadaptierte Basis für eine Erweiterung der Befunderstellung über diese Mindestanforderungen hinaus.

## Fazit für die Praxis

- Der radiologische Befundbericht stellt ein zentrales Element der interdisziplinären Kommunikation im Rahmen der Patientenversorgung dar.
- Um dieser Aufgabe optimal gerecht zu werden, sollte der Befundbericht den spezifischen Informationsbedarf der ärztlichen Zuweiser umfassend abdecken.
- Im Rahmen einer bundesweiten Expertenumfrage wurde dieser Informationsbedarf für MRT-Befunde von Patienten mit Gliomen ermittelt.
- Von neuroonkologischer Seite bestehen klare inhaltliche Ausgestaltungswünsche für die MRT-Befundung hirneigener Tumoren.
- Die exakte anatomische Tumorausbreitung inklusive ependymaler und meningealer Beteiligung sowie Zeichen der Raumforderung bilden wesentliche Kernelemente des MRT-Befunds.
- Die Ergebnisse der Studie können eine Orientierungshilfe für den Radiologen bei der Befundung dieser Pathologien bieten.

## Abkürzungen

BT-RADS: Brain Tumor Reporting and Data System
KI: 95 %-Konfidenzintervall
MRT: Magnetresonanztomographie
RANO: Response Assessment in Neuro-Oncology
ZNS: Zentrales Nervensystem
